# Supplementary figures and images for: Novel method to investigate thermal exchange rates in small, terrestrial ectotherms: A proof-of-concept on the gecko Tarentola mauritanica
Source: PLoS One. 2024 Dec 26;19(12):e0316283. doi: 10.1371/journal.pone.0316283 (PMC11670986; doi:10.1371/journal.pone.0316283)

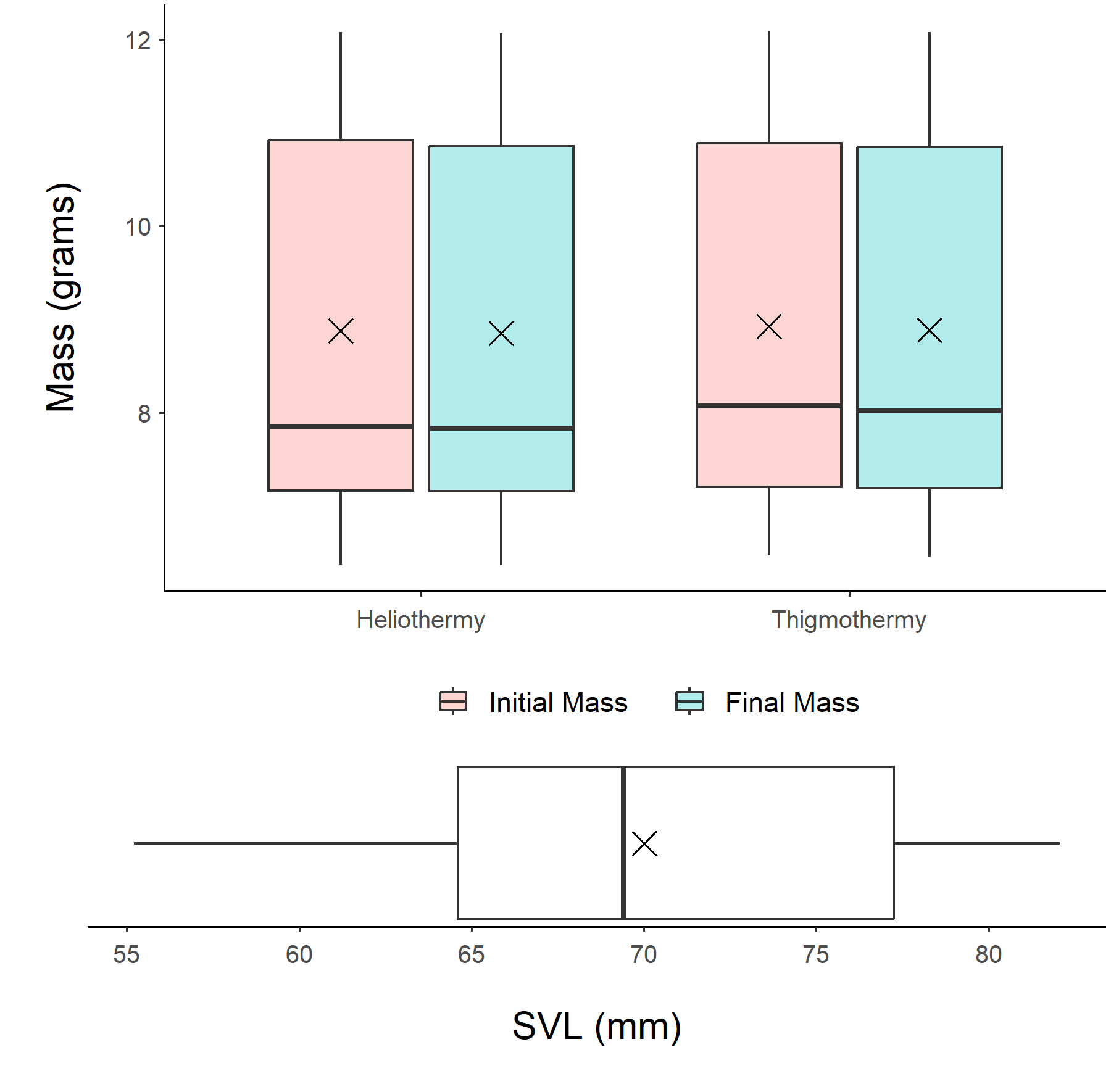

Supplement: S1 Fig — The line in the middle of the boxplot represents the median value, while the “X” represents the mean value. No significant differences between initial and final mass were observed nor were there any differences in mass between treatments. (TIF) [file pone.0316283.s001.tif]

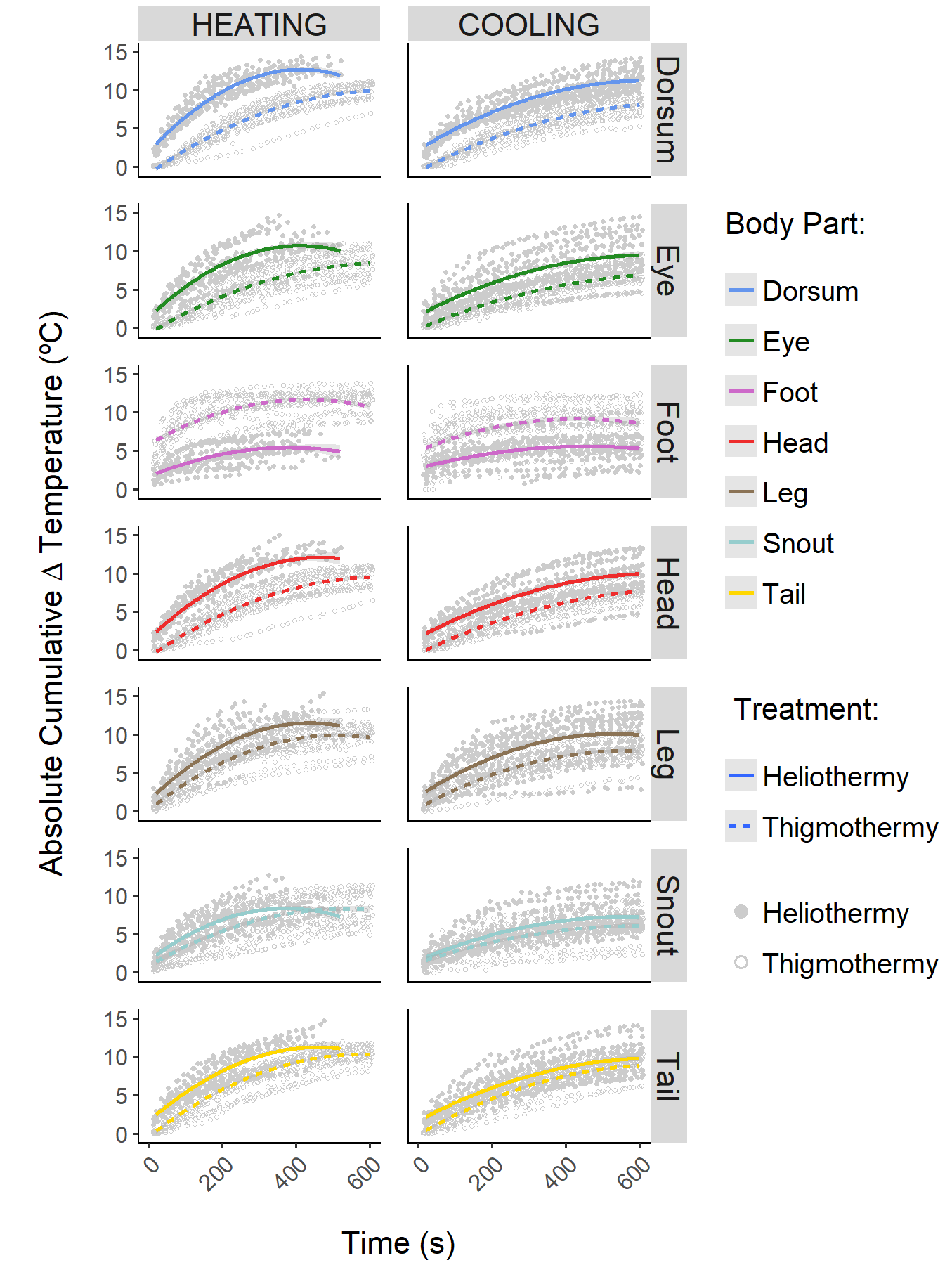

Supplement: S2 Fig — Heating (left column) and cooling (right column) profiles for the different body parts, under heliothermic (full line, full points) and thigmothermic (dashed line, empty points) treatments. 2nd order polynomial fitted to demonstrate the trend in the data. (TIF) [file pone.0316283.s002.tif]

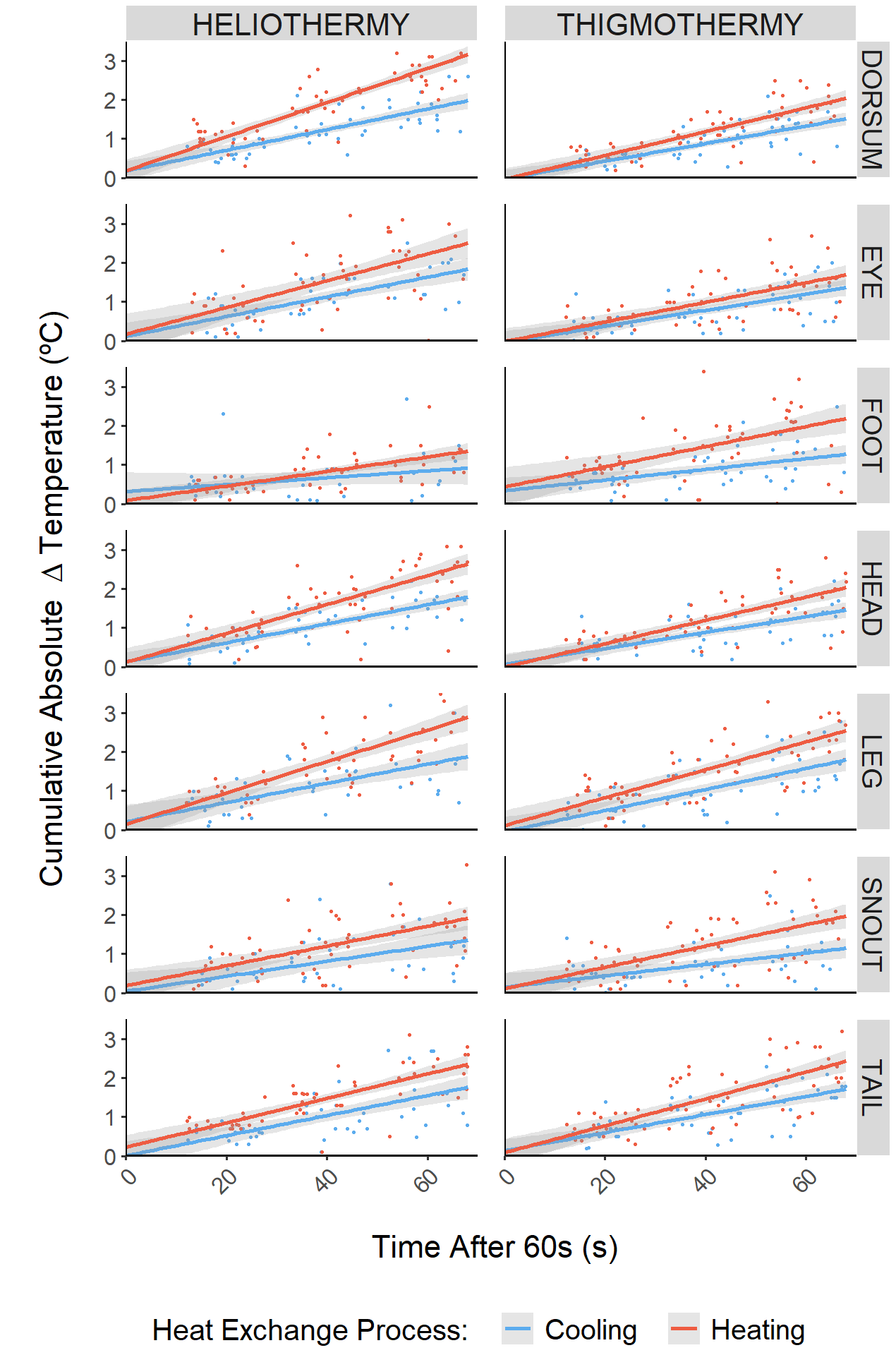

Supplement: S3 Fig — Absolute rates are plotted in order to facilitate the comparison between the magnitude of the slope between the heating and cooling processes. (TIF) [file pone.0316283.s003.tif]

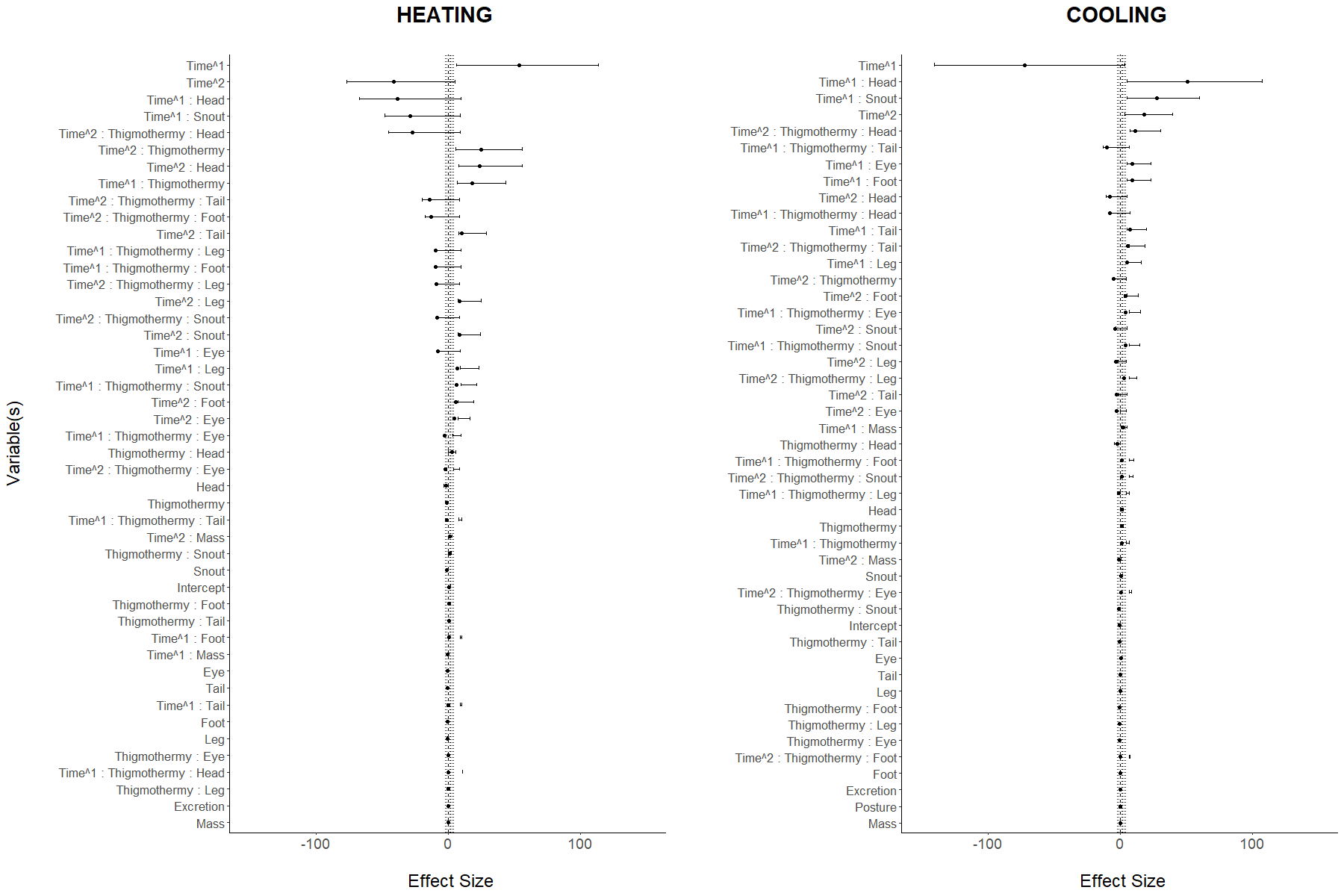

Supplement: S4 Fig — (TIF) [file pone.0316283.s004.tif]

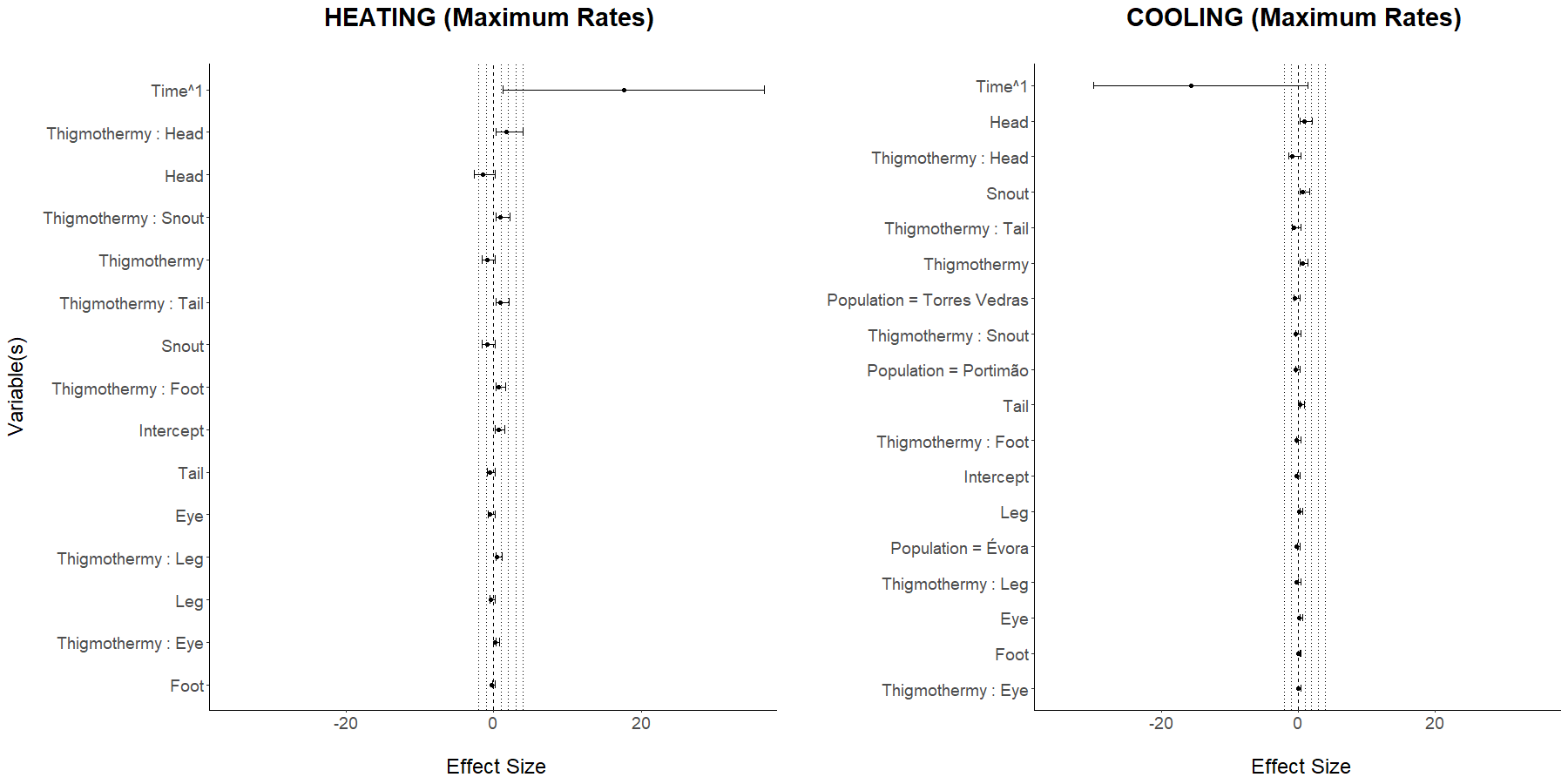

Supplement: S5 Fig — (TIF) [file pone.0316283.s005.tif]
